# Peripheral Nerve Reconstruction after Injury: A Review of Clinical and Experimental Therapies

**DOI:** 10.1155/2014/698256

**Published:** 2014-09-03

**Authors:** D. Grinsell, C. P. Keating

**Affiliations:** Plastic and Reconstructive Surgery Unit, St. Vincent's Hospital, 41 Victoria Parade, Fitzroy, Melbourne, VIC 3065, Australia

## Abstract

Unlike other tissues in the body, peripheral nerve regeneration is slow and usually incomplete. Less than half of patients who undergo nerve repair after injury regain good to excellent motor or sensory function and current surgical techniques are similar to those described by Sunderland more than 60 years ago. Our increasing knowledge about nerve physiology and regeneration far outweighs our surgical abilities to reconstruct damaged nerves and successfully regenerate motor and sensory function. It is technically possible to reconstruct nerves at the fascicular level but not at the level of individual axons. Recent surgical options including nerve transfers demonstrate promise in improving outcomes for proximal nerve injuries and experimental molecular and bioengineering strategies are being developed to overcome biological roadblocks limiting patient recovery.

## 1. Introduction

Twenty million Americans suffer from peripheral nerve injury caused by trauma and medical disorders [1]. Nerve injuries result in approximately $150 billion spent in annual health-care dollars in the United States [2]. The majority of peripheral nerve injuries occur in the upper limb and are from traumatic causes [3]. These injuries disproportionately afflict young healthy civilians and military officers who are most at risk of traumatic injuries [3]. Severe nerve injury has a devastating impact on a patients' quality of life. Typical symptoms are sensory and motor function defects that can result in complete paralysis of the affected limb or development of intractable neuropathic pain [4]. Nerve fibres of the transected nerve regenerate spontaneously to the extent limited by the size of the nerve gap, neuroma, and scar tissue formation [4]. Many injuries require surgical nerve reconstruction and a meta-analysis in 2005 [5] of median and ulnar nerve repairs demonstrated that only 51.6% achieve satisfactory motor recovery (M4-5), with even less (42.6%), experiencing satisfactory sensory recovery (S3+ to S4). Younger age and more distal injuries have better outcomes, although many articles report higher rates of “good” motor outcomes using a lower cutoff (M3—movement against gravity only). The primary goal of nerve repair is to allow reinnervation of the target organs by guiding regenerating sensory, motor, and autonomic axons into the environment of the distal nerve with minimal loss of fibres at the suture line [6].

Aegineta et al. (626–696 AD) are the first physicians who postulated the restoration of severed nerves [7]. In 1850 Waller described loss of the distal axonal segment in frog glossopharyngeal and hypoglossal nerves after injury [8]. This process of Wallerian degeneration still remains the major biological roadblock to rapid and complete nerve regeneration in mammalian nerves. In 1873 Huenter first described an epineural nerve repair technique, which remains in use today [9]. In 1892 Cajal discovered that neurotropic factors preferentially guide regenerating axons distally toward the target organ [10]. In 1945 Sunderland described the principles of microsurgical nerve repair and Kurze and Smith applied this independently in 1964 after the development of the operating microscope [11–13]. Only minor refinements in surgical technique have been made in the past 50 years and epineural repair remains the gold standard surgical reconstruction, with direct end-to-end nerve repair, or where there is excessive tension, by using interposition autologous nerve grafts.

## 2. Peripheral Nerve Anatomy

The peripheral nervous system is composed of motor and sensory neurons with their cell bodies in the spinal cord and long cytoplasmic extensions called axons, which signal with a distant target organ.

**Figure 1:**
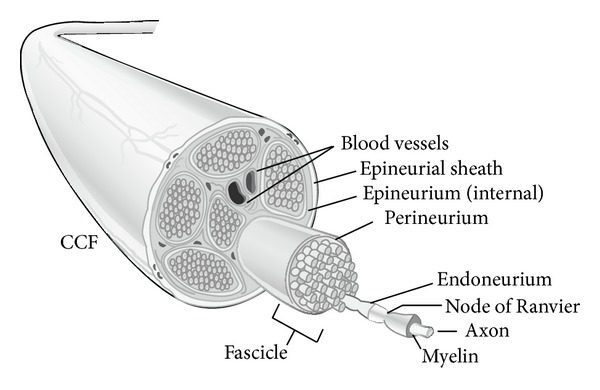
Peripheral nerve anatomy [4].

Axons are grouped together in spatially arranged motor or sensory bundles called fascicles (Figure 1). Individual axons are surrounded by a connective tissue layer, the endoneurium, and fascicles are separated by perineurium. Sunderland in 1945 [16, 17] made anatomical maps for the motor or sensory fascicle patterns within major limb nerves. These patterns are most prominent distally in a peripheral nerve where it is important to correctly match fascicles during nerve repair for optimal regeneration. Groups of fascicles are contained within a peripheral nerve surrounded by a connective tissue layer called the epineurium. The internal epineurium separates fascicles and the external epineurium surrounds all the fascicles and defines the nerve anatomically. The epineurium is sutured in nerve repair and nerve grafting and comprises 50% of the total cross sectional area of a peripheral nerve [18]. External to this layer is the mesoneurium, containing the blood supply to the nerve. A fine network of capillaries exists at the endoneurial level. This fragile blood supply is easily disrupted due to trauma or tension at the nerve repair. Therefore, nerve grafts have better outcomes than direct repair under tension, due to devascularization of the nerve [4].

## 3. Nerve Physiology

The internal neuronal environment like all cells is in carefully controlled electrolyte homeostasis. Antegrade and retrograde axoplasmic transport cycles neurotransmitters and structural cell elements back and forth between the cell body and axonal tip. Any break or defect in the axonal or neuronal bilayer lipid membrane unless rapidly repaired results in an irreversible cascade of programmed cell death [19].

**Figure 2:**
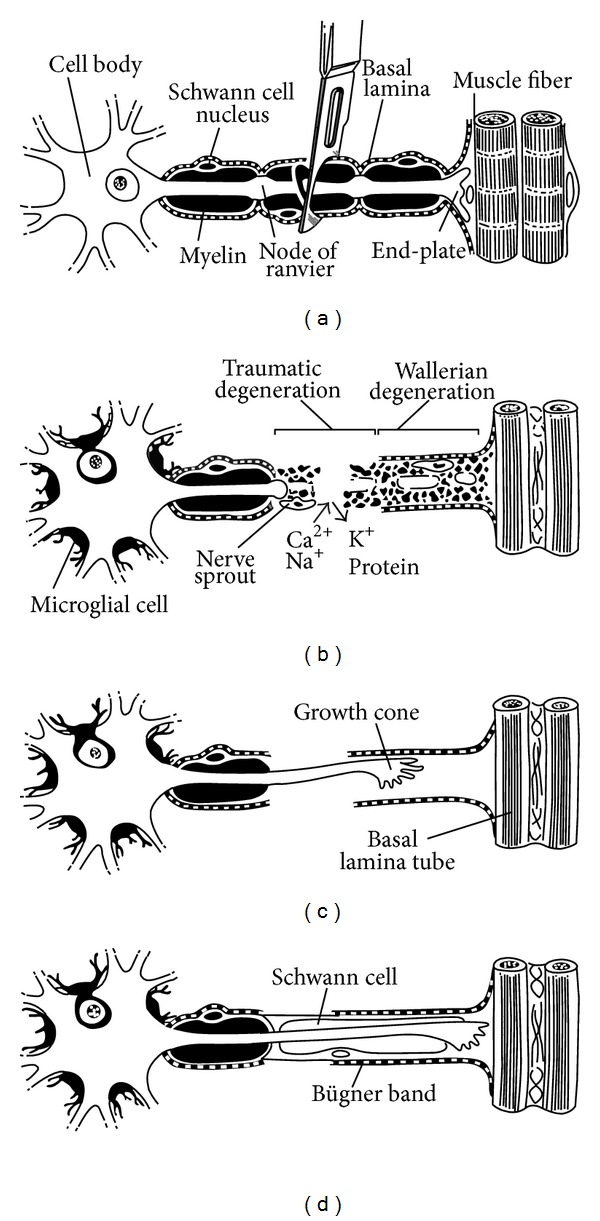
Degeneration and regeneration after peripheral nerve injury [24].

Axonal degeneration follows a sequence of events within the zone of trauma extending both proximally and distally (Figure 2). Disconnected axons and cell bodies (in proximal axon injuries) degenerate via a programmed cell death pathway called chromatolysis [20, 21]. This focal degeneration is similar to what occurs in other traumatized tissues including skin and muscle [22]. However, the major difference compared to other tissues is that Wallerian degeneration of the distal axonal segment then occurs from the zone of trauma to the motor or sensory receptor some distance away. Wallerian degeneration ensues 24–48 hours after peripheral nerve injury and both the distal axons and surrounding myelin degenerate [23]. The proximal axonal segment also degenerates back to the adjacent node of Ranvier, the site of subsequent axonal regrowth.

Schwann cells phagocytose axonal and myelin debris until empty endoneurial tubes remain. Macrophages are recruited to the area releasing growth factors, which stimulate Schwann cell and fibroblast proliferation. Schwann cells fill the empty endoneurial tubes in organized longitudinal columns called bands of Bungner [20]. This supportive environment is critical for successful axonal regeneration.

Axonal regeneration occurs from the most distal node of Ranvier. As many as 50–100 nodal sprouts appear, mature into a growth cone, and elongate responding to directing signals from local tissue and denervated motor and sensory receptors (neurotrophic and neurotropic factors) [24]. Ramon y Cajal described neurotropism in classic studies where they demonstrated that axon regrowth is directed selectively towards the distal nerve stump [10]. Further work using Y-chambers demonstrated that axons preferentially grow towards nervous tissue [14, 25, 26]. In addition, there are motor axon-motor receptor and sensory axon-sensory receptor specificity in regrowth [6, 27].

**Figure 3:**
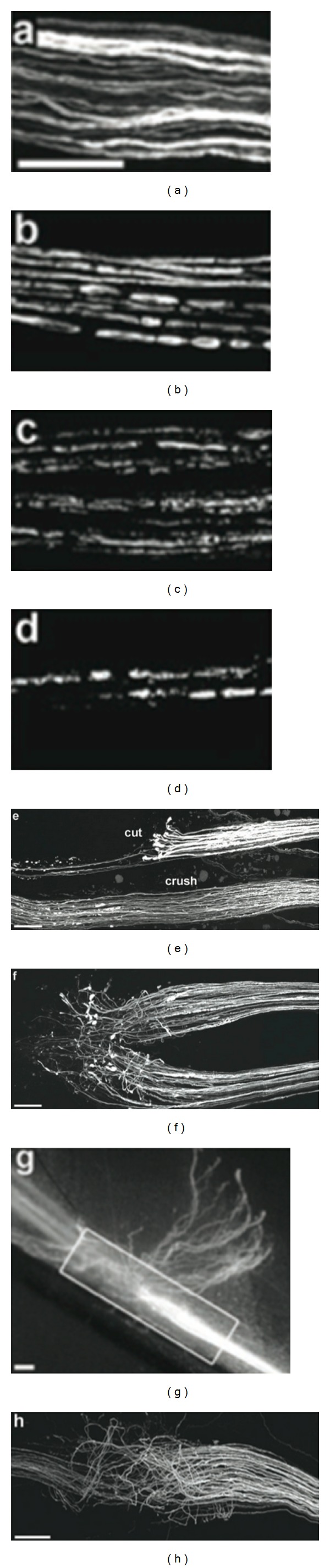
Axon regeneration after axonotmesis and neurotmesis injuries [15].

Proteases are also released from the growth cone to aid axonal regeneration through tissue. Numerous axonal extensions elongate from the growth cone until they connect with a receptor. Axonal pruning then occurs with the remaining neurites. If a receptor or endoneurial tube is not reached, growth cone branches continue to grow in a disorganized manner producing a neuroma, which can manifest clinically as a painful lump [4]. Studies by Lichtman have demonstrated that axonal regeneration is increasingly disordered with more severe nerve injury (Figure 3) [15]. This results in less axons reaching the distal sensory or motor target due to increased scarring and less effective axonal regeneration after severe nerve injury.

Denervation of sensory or motor targets due to peripheral nerve injury shrinks the cortical representation of that region in the ipsilateral brain hemisphere. Adjacent regions in both the ipsilateral and contralateral hemisphere enlarge to compensate for the injury [28]. Stimuli can also become misinterpreted between injured and uninjured tissues resulting in phantom limb and neuropathic pain.

## 4. Grading Nerve Injury

The earliest classification of nerve injury was made by Seddon in 1947 who described three injury grades (Table 1) [29]. Neurapraxia is segmental myelin damage with an intact axon, usually caused by compression. There is a temporary focal conduction block that resolves completely within 12 weeks once myelination is restored. Axonotmesis from a crush mechanism is axonal injury where the connective tissue and nerve continuity remain intact. Wallerian degeneration ensues and slow axonal regeneration follows at a rate of 1 mm/day. Incomplete recovery is common, depending on the distance for regeneration between the injury and target tissue. Neurotmesis is complete physiological and anatomical transection of both axons and connective tissue. A neuroma may form but no spontaneous regeneration occurs without surgical intervention.

Sunderland in 1951 expanded the classification based on histology to include five injury grades, which broadly correspond to Seddon's three-level classification but with more accurate prognosis of outcomes in axonotmesis injuries [13, 30]. Sunderland grades I and II recover completely, grade III recover partially, and grades IV and V usually require surgical intervention. Sunderland grade I injuries are equivalent to neurapraxia. Sunderland grade II injuries have axonal damage but intact endoneurium and hence achieve full recovery. Sunderland grades III and IV will heal spontaneously with increasing degrees of scarring and incomplete recovery due to progressive damage to axons and connective tissue (endoneurium, or endo/perineurium). Scar creates a conduction block and if severe requires excision and nerve reconstruction. Sunderland grade IV injuries usually require surgery due to damage to both axons and all levels of connective tissue (endo/peri/epineurium) with resultant extensive scarring. Sunderland grade V injuries correspond to neurotmesis.

This classification has somewhat limited clinical utility as most nerve injuries are of mixed grade and there is no diagnostic test to discriminate between Sunderland grades II and IV. Currently these Sunderland grades can only be diagnosed histologically [20]. Mackinnon and Dellon modified Sunderland's classification to include a mixed injury pattern better reflecting clinical practice (grade VI) [31].

Nerve conduction studies (NCS) and electromyograms (EMG) are noninvasive tests that have a diagnostic role in the delayed setting (six weeks later), when fibrillations in denervated muscle are present, but not immediately after injury. Therefore, there is no noninvasive diagnostic test that can diagnose the presence or severity of a nerve injury in the first six weeks after injury. Diagnosis still relies on clinical examination and/or surgical exploration.

NCS assesses both motor and sensory function via a voltage stimulator applied to the skin over different points of the nerve to be tested. The evoked response is recorded from a surface electrode overlying the muscle belly (motor response) or nerve (sensory response).

EMG assesses only motor function and consists of inserting a needle into a muscle to assess resting electrical activity (the presence of abnormal spontaneous activity such as fibrillations and positive sharp waves) and voluntary motor unit analysis [32]. Fibrillations from denervated muscle may not be apparent until three to six weeks after injury, depending on how proximal the nerve injury is [33]. Therefore, the optimal diagnostic timing of EMG will depend on the injury site.

NCS is used initially as a screening test for the presence or absence of conduction block and the addition of EMG provides valuable information in the form of reduced action potentials [23].

NCS and EMG obtained serially over time may map nerve recovery and identify a neurapraxic or axonotmetic lesion. The lack of spontaneous clinical or NCS/EMG recovery after three- to six months warrants surgical exploration depending on the level of injury. The problem remains that the most opportune time for surgical intervention has passed by then. The effects of chronic axotomy and muscle denervation render the tissue environment suboptimal for successful axonal regeneration. Since acute repair leads to better functional restoration, delays introduced by “wait-and-see” diagnostics can be costly [20]. There is a great clinical need for accurate nerve injury diagnostics in the setting of acute injury.

## 5. Timeframe for Functional Recovery

Mackinnon demonstrated that early nerve repair results in improved functional outcomes [34]. However, despite optimal nerve repair, the rate of axonal regeneration is slow at 1-2 mm/day [20]. No therapeutic methods have been devised to speed this rate of regeneration. There is an accepted window period of 12–18 months for muscle reinnervation to occur in order to achieve functional recovery before irreversible motor end plate degeneration occurs. Although there is no definite evidence to support this and the senior author has personally seen regeneration as late as 26 months after injury and reconstruction [24]. The timeframe for sensory reinnervation is longer but not infinite. A combination of slow axonal regeneration, structural changes in muscle targets, and an increasingly less supportive stromal environment for regeneration all contribute to incomplete functional recovery.

At the wrist, for example, median and ulnar nerve injuries involve distances of approximately 100 mm over which axons must regenerate to reach many of the hand muscles. At the average regeneration rate of 1 mm/day in humans, recovery requires at least 100 days. More proximal nerve injuries, such as a brachial plexus injury, involve distances of up to a metre and require periods of more than 2-3 years for regenerating axons to reach and reinnervate the hand muscles. In such cases, it is well recognized clinically that there may be little or no restoration of function. During this long period of time, neurons remain without target connections (axotomized), and the target organ and distal nerve remain denervated until reached by regenerating axons [20].

Muscle fibrosis and atrophy begins immediately after denervation and plateaus after four months when 60–80% of muscle volume has been lost [24]. Motor endplates actually increase within muscle but functional reinnervation is unlikely beyond 12 months due to the progressive fibrosis [24].

Although the failure of functional recovery has historically been attributed predominantly to irreversible atrophy of muscle targets and their replacement by fat, animal experiments are now indicating that it is the progressive failure of the neurons and Schwann cells to sustain axon regeneration over distance and time. In 1995, Fu and Gordon used a rat tibial nerve model to demonstrate that, after delayed repair of more than four months, regeneration declined to ~33% of the number of axons that could regenerate after an immediate nerve repair [35, 36]. Although muscle function was equivalent despite denervation, this was due to a smaller pool of regenerated axons compensating by innervating 3-fold the number of muscle fibres compared to normal. With increasing denervation times, the pool of successfully regenerated axons dwindled and overall muscle function declined [35, 36].

The timeframe for reinnervation of sensory receptors is much longer than that for motor nerves but earlier repair still results in better sensory outcomes [24]. Sensory receptors can be reinnervated years after injury but the maximum timeframe remains uncertain.

In summary, axon regeneration after peripheral nerve injury progressively fails due to chronic axotomy of the neurons and chronic Schwann cell denervation and is not due solely to irreversible atrophy of muscle as was previously believed [20].

## 6. Nerve Repair

**Figure 4:**
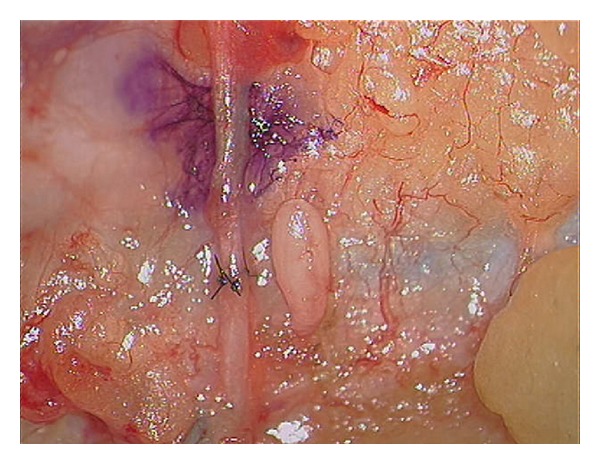
Epineural repair as seen through the microscope.

Direct nerve repair with epineural microsutures is still the gold standard surgical treatment for severe axonotmesis and neurotmesis injuries (Figure 4). Epineural repair is performed when a tension free coaptation in a well-vascularised bed can be achieved. Gross fascicular matching between the proximal and distal nerve ends results from lining up both the internal nerve fascicles and the surface epineural blood vessel patterns.

Other repairs include grouped fascicular repair requiring intranerve dissection and direct matching and suturing of fascicular groups. This is more practical distally in a major peripheral limb nerve. However, the theoretical advantages of better fascicle alignment with this technique are offset by more trauma and scarring to the healing nerve internally due to the presence of permanent sutures. Despite its anatomical attractiveness, overall group fascicular repair is no better than epineural repair in functional outcomes [37].

**Figure 5:**
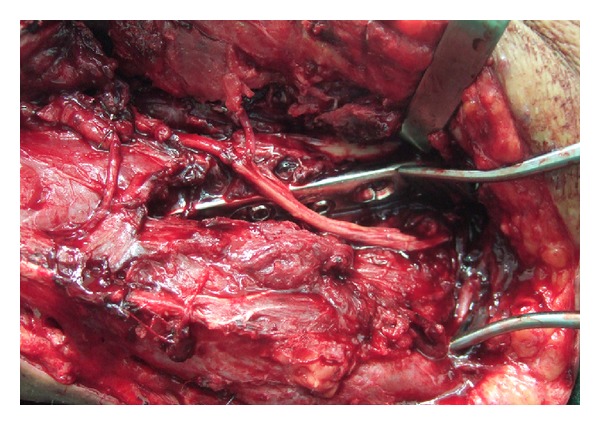
Nonvascularised cable nerve graft to reconstruct 15 cm defect of radial nerve.

When there is a gap between the nerve ends with excessive tension for direct epineural repair, reversed interposition autologous nerve grafts are required (Figure 5). Human autografts are preferred as the literature is clear that autografting is superior to nerve conduits for longer gaps (>3 cm), more proximal injuries, and critical nerves [20]. Nerve grafts can be single, cable, trunk, interfascicular, or vascularized [38]. A single graft joins nerve gaps with a segment of a donor nerve of similar diameter. To span gaps between large diameter nerves, cable grafts are used, comprising multiple lengths of a smaller diameter donor nerve to approximate the diameter of the injured nerve. Donor nerve grafts are harvested from expendable sensory nerves including the sural and medial antebrachial and are reversed in orientation to maximize the number of axons successfully regenerating through the graft by funneling them distally. This prevents loss of regenerating axons down side branches of the donor nerve graft.

Trunk grafts use a donor segment from a large nerve interposed to repair a gap in a proximal nerve. There has been poor success with this method as large diameter donor nerves fibrose internally due to poor vascularity before axons are able to regenerate across the graft [38].

The interfascicular nerve graft was described by Millesi et al. [39]. Strands of the grafted nerves are interposed between carefully dissected groups of fascicles in the damaged nerve, creating direct pathways between fascicles for regeneration.

**Figure 6:**
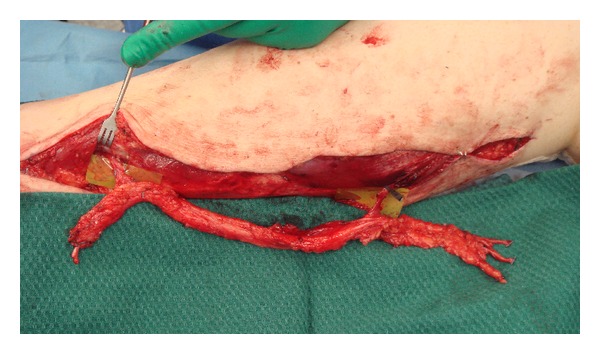
Vascularised sural nerve graft insitu before transfer.

The vascularised nerve graft was designed by Taylor and Ham, whereby the donor nerve is transposed with its arterial and venous supply into the graft site [40]. Vascularisation allows a nerve graft to avoid the initial period of ischaemia and ensures continuous nutrition of the graft (Figure 6). Intraneural fibrosis is avoided and axonal regeneration and target connectivity is enhanced [41]. Terzis and Kostopoulos clinically demonstrated that medium-sized trunk grafts, which would normally undergo central necrosis, could be transferred as vascularized nerve grafts and survive [41]. The clinical indication for a vascularized nerve graft is a scarred recipient bed that will not support a nonvascularized nerve graft [41].

The harvested autologous nerve graft undergoes Wallerian degeneration and thus merely provides mechanical guidance creating a supportive structure for the ingrowing axons [42]. Autologous nerve grafts fulfill the criteria for an ideal nerve conduit because they provide a permissive and stimulating scaffold including Schwann cell basal laminae, neurotrophic factors, and adhesion molecules [4].

However, autografts sacrifice a functioning nerve (sensory), for a more important injured nerve (usually motor). There is sensory loss and scarring at the donor site and potential for neuroma formation [43]. At the repair site there is unavoidable size and fascicle mismatch, scarring and fibrosis from sutures, tissue handling, and the injury itself and all of these factors lead to poor regeneration. A clinical rule of thumb is that there is a 50% loss of axons at each coaptation site. Therefore, for primary nerve repair, approximately 50% of the original axons will successfully regenerate through the repair site. For a nerve graft with two coaptation sites, 25% of axons will successfully regenerate through the graft. Depending on the distance to the motor/sensory target, there will then be additional axonal loss due to the effects previously discussed of chronic axotomy and muscle fibrosis.

Human cadaveric nerve allografts have been used in a limited number of patients with extensive nerve injuries and inadequate autologous nerve donor tissue. Compared to autografts there are no donor supply limitations or donor site morbidity; however, there are significant costs and complexity with their use [43]. Donor Schwann cells display major histocompatibility complexes and incite a T-cell response [23]. Therefore, recipients are immunosuppressed for up to two years until the donor nerve graft has been repopulated with host Schwann cells. Moore et al. state that nerve allotransplantation should be reserved for unique patients with irreparable peripheral nerve injuries, which if left untreated, would lead to an essentially nonfunctional limb [43].

Nerve allografts have also been decellularized by a process of chemical detergent, enzyme degradation, and irradiation resulting in a graft with no requirements for immunosuppression [44]. The advantage of these clinically available grafts (AxoGen), over hollow nerve conduits, is that the internal nerve structure including endoneurial tubes, basal lamina, and laminin remain intact, facilitating axonal regeneration [44]. A recent level III study demonstrated functional recovery for injuries with gaps between 5 and 50 mm [45]. However, currently their use like hollow conduits is limited to small sensory nerves, for example, digital nerves, for gaps less than 3 cm. Decellularized nerve grafts or nerve conduits are not considered a replacement for autologous nerve grafting in motor nerves, gaps more than 3 cm, or in proximal nerve injuries.

Numerous conduits have been described but none of these have demonstrated equivalent or superior outcomes to autografts for gaps greater than 3 cm. Conduits can be categorized as autogenous biological, nonautogenous biological, or nonbiological [38]. Autogenous biological conduits include hollow vein and arterial conduits and soft tissues, including muscle and tendon grafts [46]. Arterial and tendon conduits have not been used clinically. The concern with muscle grafts is that regenerating axons are not contained within the graft and may form neuromas or aberrant regeneration. Vein conduits are the most popular biological conduits and Chiu and Strauch conducted a prospective study of twenty-two patients with defects of <3 cm in the hand and forearm, finding that autogenous vein nerve conduits produced results comparable to sural nerve digital grafts [47]. The use of vein grafts is usually reserved for small, less functional nerves with small nerve gaps (e.g., digital sensory nerves with less than a 3 cm gap).

Nonautogenous biological conduits have been made from collagens type I, III, or IV and are available clinically. Animal studies with collagen conduits have demonstrated equivalent efficacy when compared with autograft; however, clinical studies are lacking [23].

Modern second generation resorbable nonbiological conduits are made from polyglycolic acid (PGA), polylactic acid (PLA), or poly lactide-co-glycolide acid (PLGA) [46]. Nonresorbable conduits including silicone and Gore-Tex demonstrated unwanted effects including axonal compression during regeneration and fibrous foreign body reaction [46]. PGA nerve conduits have been assessed by a number of clinical studies and demonstrate equivalent results to nerve repairs or autologous grafts for short or moderate digital nerve gaps [23].

All clinically available conduits are hollow tubes although extensive research continues to focus on adding internal structure, Schwann cells, and growth factors to support axonal regeneration. Rinker and Liau in their recently published prospective trial comparing vein grafts to PGA conduits in sensory nerve gaps of 4–25 mm demonstrated equivalent cost and sensory outcomes at 12 months [48]. Therefore, all autologous nerve graft alternatives including decellularized nerve grafts and autogenous and nonautogenous conduits demonstrate similar efficacy but their use is limited to sensory nerves with small gaps <3 cm. Primary nerve repair or autogenous nerve grafts remain the mainstay of surgical nerve reconstruction for severe nerve injuries.

Overall the limit of our current technical abilities with an operating microscope is to line up or directly coapt individual or groups of fascicles within a nerve. However, we cannot manipulate the behaviour of individual axons, which is governed at a molecular nanometer level [20]. Ultimately this biological hurdle accounts for the incomplete and often poor functional outcomes that occur despite our best efforts at nerve reconstruction.

## 7. Results of Nerve Repair

Functional nerve recovery relies on motor axons correctly matched to motor endplates and sensory axons reaching their sensory receptors. Most studies have graded the success of nerve repair using the British Medical Research Council's system or its modified versions for the evaluation of motor and sensory return. Physical examination allows grading of sensory recovery from S0 to S5 and motor from M0 to M5 [23]. Mackinnon and Dellon reported in a 40-year compilation of data that after direct nerve coaptation 20–40% achieved very good (M4S3+) recovery after nerve repair but that few injuries recovered fully [31].

The results of nerve grafts (and allografts) are worse than for nerve coaptation. Grafts proximal to the elbow, more than 7 cm in length, older patients, and greater delay to nerve reconstruction are adverse prognostic features [24].

No alternatives to autologous nerve grafts have demonstrated equivalent outcomes in gaps >3 cm. For small gaps, the application of artificial resorbable nerve guides to bridge nerve defects up to 3 cm has the same success rate as nerve autograft repair, which results in recovery in up to 69% of cases [4].

In 1990 Sunderland summarized 40 years of clinical experience in nerve repair: early repairs are better than late; nerve coaptation is better than nerve grafts; young do better than old; distal repair is better than proximal repair; short grafts do better than long [49]. These principles remain equally as relevant today.

## 8. Surgical Alternatives to Nerve Repair: Nerve Transfers and Free Functioning Muscle Transfer

**Figure 7:**
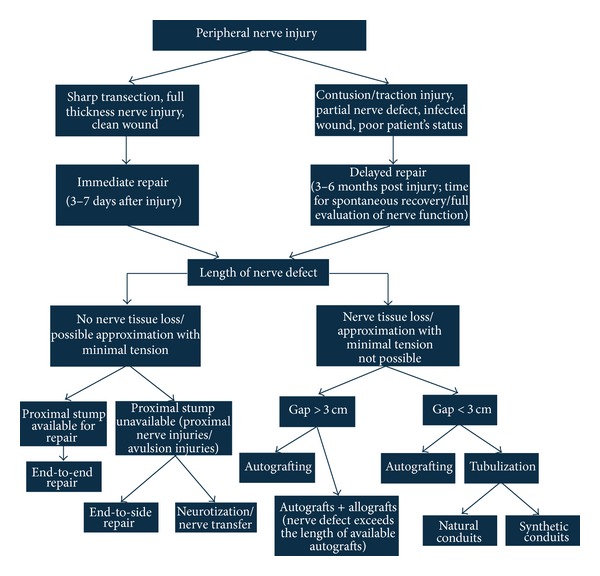
Surgical algorithm of peripheral nerve repair [4].

Alternate strategies exist to bypass injured peripheral nerve pathways using healthy donor nerves (Figure 7). This is indicated in very proximal nerve injuries or in those without a proximal nerve stump, for example, cervical nerve root avulsions.

The definition of nerve transfer is the surgical coaptation of a healthy nerve donor to a denervated nerve [50]. This is usually reserved for important motor nerve reconstruction although it can equally be applied to critical sensory nerves. Nerve transfers use an expendable motor donor nerve to a less important limb muscle. The nerve is cut and then joined to the injured distal end of the prioritised motor nerve.

As early as 1921, Harris described a radial to median nerve transfer to treat a low median nerve injury suffered during World War I [51]. However, in the ensuing decades particularly after the advent of microsurgery in the 1960s nerve autografting achieved success and became the preferred reconstructive method. It was not until the 1970s and 1980s that interest in nerve transfers was revived [52].

The benefits of nerve transfers are well described. In most cases there is only one neurorrhaphy site; with nerve grafts, there are two. In addition, nerve transfers minimize the distance over which a nerve has to regenerate because it is closer to the target organ and is more specific [52]. Pure motor donors are joined to motor nerves and sensory donors to sensory nerves, optimizing regeneration potential. As opposed to a tendon transfer, when a nerve transfer is successful, recovered function is similar to the original muscle function because synchronous physiologic motion may be achieved. With quicker nerve recovery, more rapid motor reeducation is also possible [50]. The goal is to maximize functional recovery with fast reinnervation of denervated motor targets [53].

**Figure 8:**
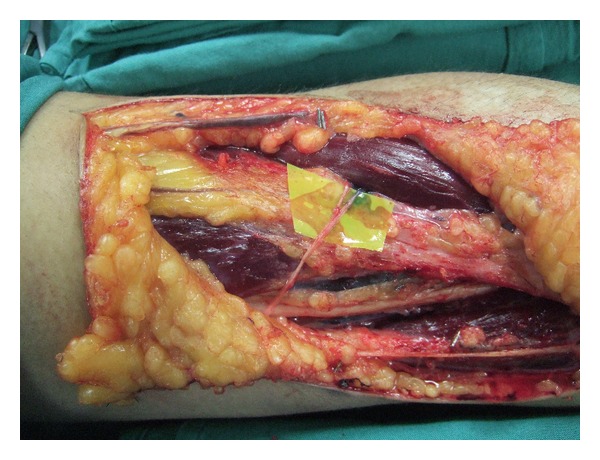
Pronator teres nerve transfer for wrist extension in radial nerve palsy. Video of pronator nerve transfer to reconstruct wrist extension (see Supplementary Material available online at http://dx.doi.org/10.1155/2014/698256).

The most common applications of motor nerve transfers include the restoration of elbow flexion, shoulder abduction, ulnar-innervated intrinsic hand function, radial nerve function (Figure 8, and Supplementary Video), and smile reconstruction from facial nerve palsy.

The disadvantages are finding an expendable donor nerve near the target muscle with a large enough motor fiber population from which to “borrow” [53]. Importantly, the donor nerve target should be synergistic with the redirected target for the brain to accommodate the rewiring of the newly redirected fibers [53].

Free functioning muscle transfer (FFMT) is another reconstructive option for severe and delayed nerve injuries including those that have failed after primary reconstruction, providing an uninjured donor nerve can be located [41]. The procedure transfers a healthy muscle and its neurovascular pedicle to a new location to assume a new function [54]. This can be used in a situation where both the nerve and muscle are damaged due to either severe acute injury or changes from chronic axotomy and muscular fibrosis. The muscle is powered by transferring a viable motor nerve to the nerve of the FFMT and restoring the circulation of the transferred muscle with microsurgical anastomosis to recipient vessels. Within several months, the transferred muscle becomes reinnervated by the donor nerve, eventually begins to contract, and ultimately gains independent function.

Function provided by the FFMT most commonly is elbow flexion but also includes elbow extension, finger and wrist extension, and grasp, for example, in cases of complete brachial plexus avulsion with limited donor nerve options for nerve transfers [55]. It is a complex procedure that should be considered only after more simple procedures are no longer options for reconstruction [54]. The senior author has pioneered a number of original procedures for reconstructing a variety of defects including reconstruction of the smile, total lip defects, quadriceps, and gluteal function.

Current indications for FFMT in brachial plexus injuries include the time when reinnervation of native musculature is not possible (i.e., traumatic loss of muscle), late reconstruction (i.e., >12 months), previously failed reconstruction, or acute injuries to restore prehension [54]. Several authors have reported good to excellent results from transfer of a single gracilis muscle for elbow flexion. In Carlsen et al.'s. experience, 79% of patients who undergo a single gracilis muscle transfer for elbow flexion alone experienced M4 strength or better [54]. Dodakundi et al. in 2013 reported long-term outcomes for 36 double free muscle transfers to restore composite upper limb function after total brachial plexus injury [55]. 70% of patients achieved M4 elbow flexion, with an average total active motion of the fingers of 46 degrees. Importantly, 48% of patients used their injured hand in activities of daily living.

## 9. Translational Research in Peripheral Nerve Repair

Research strategies to improve recovery after nerve repair fall into two main categories: methods that enhance axonal regeneration and methods that decrease environmental inflammation. Methods to enhance axonal regeneration can be further broken down into (Section 9.1) enhancing axonal sprouting from the distal nerve stump (growth factors; electrical stimulation of the proximal stump); (Section 9.2) providing a permissive environment for axons to cross a coaptation (enhanced conduits; thermal and nonthermal laser; nerve glue) (Section 9.3); delaying or altering Wallerian degeneration (axon fusion); (Section 9.4) shortening the denervation time of muscle (electrical stimulation of the motor target).

### 9.1. Enhancing Axonal Regeneration

#### 9.1.1. Growth Factors

Nerve growth factors (neurotrophins) are molecules that are naturally released in the process of nerve regeneration. They are released from the nerve ending especially following a nerve injury and have an effect on nerve growth, differentiation, and surveillance [46]. A number of these neurotrophic factors have been isolated and applied to the proximal nerve stump after injury to enhance axonal regeneration.

Nerve growth factor (NGF) is present at low concentrations in healthy nerves. Following nerve injury, NGF is upregulated in the distal nerve stump and plays an important role in the survival of sensory neurons and outgrowth of their neurites [46]. There are numerous other growth factors that have been identified during nerve regeneration including Glial growth factor (GGF), fibroblast growth factor (FGF), glial cell derived neurotrophic factor (GDNF), neurotrophin 3 (NT-3), ciliary neurotrophic factor, and leupeptin [24, 46].

NGF, GGF, GDNF, and NT-3 have been applied in nerve conduits to small animal models of nerve gap injury (1–4 cm gap), demonstrating improved histological, electrophysiological, and functional outcomes compared to conduit controls [46]. However, one of the few studies comparing NGF seeded conduits versus nerve autografts demonstrated superior functional outcomes in the autograft group [56]. Future application of growth factors in combination or via sustained release delivery systems or scaffolds could further enhance axonal regeneration, particularly for conduits in nerve gap injuries.

#### 9.1.2. Electrical Stimulation

There have been limited reports of applying electrical fields/gradients across a repaired peripheral nerve to speed up axonal regeneration. Animal studies demonstrate that as little as one hour of direct nerve electrical stimulation immediately after repair of a transected femoral nerve in the rat promotes a dramatic increase in the kinetics of target muscle reinnervation [57].

In a clinical pilot study, one hour of electrical stimulation was applied after median nerve decompression at the wrist for 21 patients with carpal tunnel syndrome and thenar atrophy [58]. The electrical stimulation group showed evidence of accelerated axonal regeneration and target reinnervation through motor unit number estimation and sensory and motor nerve conduction studies.

### 9.2. Optimising Axonal Regeneration across a Coaptation

#### 9.2.1. Nerve Conduits

Significant research has gone into methods that are alternatives for nerve grafts, with most efforts focused on developing improved nerve conduits with internal structure, neurotrophic factors, or Schwann cells.

The ideal synthetic conduit should be permeable enough to provide sufficient diffusion of oxygen and metabolites for supporting Schwann cells proliferation but should also prevent fibroblast infiltration [59]. Schwann cell migration into nerve conduits or acellularized allografts is insufficient beyond 2 cm and is therefore one of the major limiting factors to axonal advancement over large gaps [20].

The engineering challenges for nerve repair are to accommodate larger deficits (diameter and length), maximise the number of regenerating axons, and guide axons with target specificity. An effective nervous tissue construct may require some combination of three primary components: a scaffold, cells, and signaling factors. Scaffolds provide a temporary structure necessary for Schwann cell migration and axon outgrowth and are eventually replaced with host cells and extracellular matrix [20].

Different growth factors can be incorporated directly (in solution), into the tube's lumen or through a delivery system. Because the effect of growth factors is often dose-dependent and requires their release over extended periods, delivery systems are generally preferred [46].

The results of growth factor enhanced conduits remain inferior to nerve autografts as previously described. In addition, many conduit luminal scaffolds have been attempted, from collagen and laminin hydrogels to synthetic and collagen filaments and channels. However, these modifications have not produced results better than an autograft either and therefore they do not offer a substantial benefit over the autograft at this time [20].

#### 9.2.2. Nonthermal Laser Amnion Wrap

Photochemical tissue bonding (PTB) creates a covalently bonded nerve wrap around a nerve coaptation, using an Nd/YAG laser, photoactive dye, and a nonimmunogenic amnion wrap [60–63]. The problems of unintended thermal injury to nerve tissue from traditional laser techniques are avoided. Collagen fibres in the amnion wrap are covalently bonded to collagen in the epineurium. This bond adds strength to the repair, concentrates neurotrophic and neurotropic factors inside the coaptation where they are needed, excludes inflammatory mediators from the extrinsic tissues, and contains regenerating axons, guiding them distally towards the motor/sensory target.

Animal studies in rat sciatic nerve and rabbit common peroneal nerve models have demonstrated improved axon counts and gait function after end-to-end coaptation with a PTB nerve wrap [60, 62, 63]. Improved gait function has also been demonstrated in a one cm rat sciatic nerve graft model [61]. To date, no clinical trials have been performed with this technique.

#### 9.2.3. Thermal Laser Welding

Thermal laser achieves tissue bonding by denaturation of structural proteins, which anneal and join when cooled. Tse and Ko reported successful nerve coaptation by laser welding in 1985; however, this was followed by reports of frequent dehiscence of 12% to 41% [64]. To prevent dehiscence, one or two stay sutures can be placed before laser welding; however, nylon stay sutures lose their tensile strength when irradiated with a CO_2_ laser [64].

Although CO_2_ laser-welded nerve adhesion has demonstrated favorable results in animal models, its clinical use can be cumbersome and its versatility is limited [64]. Concerns remain about the high rate of nerve dehiscence and thermal injury to axons and nerve tissue.

#### 9.2.4. Glue Repair

Advantages of an adhesive for nerve repair include ease of use, less tissue trauma, maintenance of nerve architecture, better fascicular alignment, and less scarring compared to microsutures [64].

The ideal glue should not induce fibrosis that can lead to nerve compression and in the case of substance interposition between nerves, it should not act as a barrier to nerve regeneration. The glue should provide adequate mechanical strength to prevent gapping or rupture at the initial repair and during the postoperative period [64].

Fibrin sealants have a proven track record as a safe and effective nerve glue [64]. The longest and greatest experience with nerve glue is in brachial plexus reconstruction. In this setting, fibrin glue has been indispensable. Narakas reported significantly reduced operative times and the ability to perform nerve repairs in areas where it was previously not possible [65, 66]. Nerve glue allows repairs to be performed at or immediately within the bony foramen of a proximal nerve root where quality suture repair is not possible [64].

A systematic review of fibrin glue for peripheral nerve repair revealed 14 animal studies, 1 cadaver study, and 1 human study that fit the study criteria [67]. Most found fibrin glue repair to be equal or superior to suture repair.

However, in clinical practice, concerns remain about the lack of adequate tensile strength for fibrin glue repair alone. A biomechanical study of rabbit sciatic nerve repair reported inferior load to failure and load to gapping with fibrin glue only relative to suture repair immediately after repair [68]. Similar inferior load to failure results have been found in a rat sciatic nerve model immediately and 7 days after repair. Fibrin glue repair was equal in strength to suture repair after a delay of 14 and 28 days [64]. Therefore, in clinical practice, fibrin glue is predominantly used as an adjunct to microsutures or to coapt nerves where suturing is not possible, for example, intervertebral foramen.

Another biocompatible glue is PEG hydrogel, which demonstrates stronger adhesion than fibrin glue without being neurotoxic [64]. In a rat sciatic nerve model, Lin and coworkers created a 5-mm nerve defect as a model of nerve coaptation under tension and repaired the nerve with 10-0 nylon epineural sutures, fibrin glue, or PEG hydrogel [69]. Nerve gapping occurred in the nerves repaired with fibrin glue but not in the suture or PEG hydrogel groups.

PEG may be superior to fibrin glue because of its greater tensile strength and longer duration before breakdown (4 weeks). PEG is nontoxic and biocompatible and does not induce a significant inflammatory response. What may be an additional advantage is that it may have adhesion-inhibiting properties that prevent perineural scarring. PEG hydrogel is therefore a promising candidate as a nerve glue [64].

### 9.3. Delaying or Altering Wallerian Degeneration

#### 9.3.1. PEG Fusion

Wallerian degeneration remains one of the major biological hurdles to rapid and complete functional reinnervation and recovery. It is well accepted that axons regenerate slowly at 1 mm/day and over large distances, functional recovery is usually incomplete. Unlike mammalian axons, peripheral nerves in invertebrates including earthworms and crayfish are able to delay or even avoid Wallerian degeneration after neurotmesis injury by reconnection of the proximal and distal axon ends [19].

Axonal membrane fusion repair utilizes the principles of hybrid cell fusion, the technique of joining the lipid membranes of two separate cells to form a single large cell, to artificially fuse mammalian axons after injury. Axonal membrane fusion joins severed axonal ends within minutes to hours after injury using the fusogen polyethylene glycol (PEG) [19].

Preliminary studies have been promising demonstrating a small improvement in gait function after nerve crush injury (axonotmesis) and a 13-fold improvement after neurotmesis injury, compared to standard of care microsurgery [70, 71]. A recent study reported that compound action potentials could be recorded distal to a 10-mm nerve graft repair up to 7 days after surgery [72]. The mechanism of this phenomenon and its implications require further investigation [64].

### 9.4. Shortening Denervation Time

#### 9.4.1. Distal Electrical Stimulation

The effects of chronic axotomy on muscles lead to irreversible fibrosis and changes that prevent successful reinnervation. Preventing or minimizing these degenerative changes during the delays caused by slow axonal regrowth could lead to improved functional outcomes. Distal electrical stimulation of muscles to maintain function is one method of achieving this aim.

Williams in 1996 reported several animal experiments on limb and facial muscle using an implantable electrical stimulator [73, 74]. In all experiments, a beneficial effect was demonstrated with improved morphology and functional capacity of the reinnervated stimulated muscles when compared with nonstimulated controls. Williams found that electrical stimulation using this implantable system could be applied for extended periods without evidence of discomfort in the experimental animals.

#### 9.4.2. Immunosuppression

FK506 (Tacrolimus) is well known to augment nerve regeneration and facilitate allografting of nerves via immunosuppression. Since composite tissue transplantation has occurred with whole hands and now faces, better than expected nerve recovery has been demonstrated with 2-point discrimination and intrinsic muscle function. This is attributed to the use of FK506 [4].

However, its use to date is restricted to uncommon situations including nerve allografts and composite tissue allotransplantation, where immunosuppression is critical to prevent tissue rejection. There is currently no role in standard peripheral nerve repair with autologous tissues.

## 10. Summary

Functional recovery after peripheral nerve repair has slowly improved since the development of microsurgical repair techniques more than 50 years ago. Nevertheless, many patients particularly with proximal nerve injuries suffer incomplete recovery and lifelong disability.

Currently there remain significant unmet needs in peripheral nerve surgery including

1. accurate diagnostics to assess nerve injury in the acute setting;
2. tissue-engineered nerve conduits that match or exceed nerve grafts;
3. clinical methods of target maintenance until reinnervation.

Direct nerve repair yields the best results and nerve autografts remain the gold standard treatment for nerve gaps. Conduits have a limited role in small gaps <3 cm for sensory nerves, but this may expand in the future with improvements in conduit design.

The biological roadblocks to early and complete recovery remain Wallerian degeneration, slow axonal regeneration, and the effects of chronic axotomy on denervated muscles. Translational research therapies address some of these barriers and future advances in surgical care may come from enhancing axonal regrowth, electrically stimulating the distal motor target after injury, and most powerfully delaying or avoiding Wallerian degeneration.

Clinical options exist for partially bypassing damaged peripheral nerve pathways using nerve transfers and free functioning muscle transfers. This paradigm of bypassing damaged nerves may be expanded in the future with experimental techniques connecting myoelectric prostheses directly to peripheral nerve stumps or even the brain.

## Supplementary Material

Video of pronator teres nerve transfer to reconstruct wrist extension in radial nerve palsy.

## Figures and Tables

**Table 1:** Nerve injury classification in increasing severity.

Sunderland [13]	Seddon [29]	Features
Type 1	*Neuropraxia *	Damage to local myelin only
Type 2	*Axonotmesis *	Division of intraneural axons only
Type 3	*Axonotmesis *	Division of axons and endoneurium
Type 4	*Axonotmesis *	Division of axons, endo- and perineurium
Type 5	*Neurotmesis *	Complete division of all elements including epineurium
Type 6∗	*Mixed *	Combination of types 2–4

*Mackinnon modification of Sunderland's criteria [15] and is a common clinical scenario.
